# Preclinical assessment of thrombin‐preconditioned human Wharton’s jelly‐derived mesenchymal stem cells for neonatal hypoxic‐ischaemic brain injury

**DOI:** 10.1111/jcmm.16971

**Published:** 2021-10-15

**Authors:** Jung‐Ho Noh, Ji‐Seong Jeong, Sang‐Jin Park, Kyung Jin Jung, Byoung‐Seok Lee, Woo‐Jin Kim, Ji‐Seok Han, Min‐Kyung Cho, Dong Kyung Sung, So Yoon Ahn, Yun Sil Chang, Hwa‐Young Son, Eun Ju Jeong

**Affiliations:** ^1^ Department of Toxicological Evaluation and Research Korea Institute of Toxicology Daejeon Republic of Korea; ^2^ College of Veterinary Medicine Chungnam National University Daejeon Republic of Korea; ^3^ Stem Cell and Regenerative Medicine Institute Samsung Medical Center Samsung Biomedical Research Institute Seoul Republic of Korea; ^4^ Department of Pediatrics Samsung Medical Center Sungkyunkwan University School of Medicine Seoul Republic of Korea

**Keywords:** disease, hypoxic‐ischaemic encephalopathy, mesenchymal stem cell transplantation, newborn, toxicology

## Abstract

Hypoxic‐ischaemic encephalopathy (HIE) is a type of brain injury affecting approximately 1 million newborn babies per year worldwide, the only treatment for which is therapeutic hypothermia. Thrombin‐preconditioned mesenchymal stem cells (MSCs) exert neuroprotective effects by enriching cargo contents and boosting exosome biogenesis, thus showing promise as a new therapeutic strategy for HIE. This study was conducted to evaluate the tissue distribution and potential toxicity of thrombin‐preconditioned human Wharton's jelly‐derived mesenchymal stem cells (th‐hWJMSCs) in animal models before the initiation of clinical trials. We investigated the biodistribution, tumorigenicity and general toxicity of th‐hWJMSCs. MSCs were administered the maximum feasible dose (1 × 10^5^ cells/10 µL/head) once, or at lower doses into the cerebral ventricle. To support the clinical use of th‐hWJMSCs for treating brain injury, preclinical safety studies were conducted in newborn Sprague‐Dawley rats and BALB/c nude mice. In addition, growth parameters were evaluated to assess the impact of th‐hWJMSCs on the growth of newborn babies. Our results suggest that th‐hWJMSCs are non‐toxic and non‐tumorigenic in rodent models, survive for up to 7 days in the brain and hold potential for HIE therapy.

## 1 INTRODUCTION

Cellular therapy refers to medical treatment that restores the functions of cells and tissues using living cells. Cell therapeutics are manufactured by physical, chemical or biological methods such as in vitro culturing, proliferating and selection of autologous cells, allogeneic cells or xenogeneic cells. Among cell therapeutics, stem cell therapeutic agent is an undifferentiated cell with the ability to differentiate into cells that make up various body tissues and can restore damaged human functions by self‐replicating, differentiating or secreting various growth factors. For the aforementioned reasons, a number of treatments that use stem cells are being developed worldwide. Further, several stem cell treatments are commercially available or under clinical trials.

Hypoxic‐ischaemic encephalopathy (HIE) is a type of brain dysfunction occurring in newborns, which is induced by oxygen deprivation and limited blood flow, known as neonatal hypoxia‐ischemia.^1^, ^2^ HIE is a major intractable developmental brain disease that accounts for 23% of all neonatal deaths and is reported to occur in 1.15 million babies annually worldwide.^3^ In the case of brain disease, severe damage from HIE results in 25%–50% of deaths. Further, 65%–75% of babies that survive can suffer permanent neurological disorders such as cerebral palsy, cognitive disorders, convulsions, learning disorders, and hearing and vision impairments.^4^, ^5^ Despite severe damage to the brain and associated mortality, there are currently no well‐established treatment strategies for HIE, except for therapeutic hypothermia.^6^ Therapeutic hypothermia reduces the extent of permanent brain damage by lowering body temperature.^7^, ^8^ However, it has been reported that the effects of therapeutic hypothermia are insignificant for severe brain damage in newborns.^9^, ^10^ However, improved therapeutic effect was confirmed when a combination treatment of stem cell transplantation and therapeutic hypothermia was used.^11^, ^12^ Therefore, the application of human umbilical cord blood‐derived stem cells can be considered as an improved therapeutic method for the treatment of HIE.

Mesenchymal stem cells (MSCs) exert their therapeutic mechanism of action through paracrine effects that regulate various functions such as cell‐to‐cell communication and immunomodulation through molecules released from the cells.^13^, ^14^ Various methods have been studied to increase the efficacy of MSCs. Previous studies have found that thrombin preconditioning of naive MSCs under certain conditions improves their paracrine property, the main mechanism of action of MSCs, without causing a loss in the biological functions of MSCs such as cell viability.^15^ Sung suggested that thrombin preconditioning improves the therapeutic properties of MSC‐derived extracellular vesicles and increases their cargo protein levels through the activation of PAR‐1 and other pathways.^15^ Kim also suggested that thrombin preconditioning significantly improves neuroprotective properties such as antioxidative, anti‐apoptotic and anticytotoxic effects in human. Wharton's jelly‐derived MSCs attenuated severe brain infarction induced by HIE with enhanced paracrine anti‐astroglial, anti‐inflammatory and anti‐apoptotic effects.^16^

However, stem cell therapy products have the risk of tumorigenicity or potential toxicity owing to their inherent property of multipotency, cell culture and other manipulations made during the manufacturing process. Hence, it is essential to ensure safety of stem cell therapy products through preclinical studies including tumorigenicity and general toxicity studies before clinical trials in humans. MSCs are known to have a relatively low risk of toxicity, such as tumorigenicity, compared to pluripotent stem cells, but the possibility of tumour growth because of their stem cell characteristics and external factors should not be excluded.^17^ This is because genetic modification caused by various culture conditions, such as long‐term culture and excessive growth promotion caused by immoderate growth factors during the manufacturing process can lead to tumour formation.^18^ Therefore, there is need for thorough evaluation of the safety of thrombin‐preconditioned MSCs obtained from an advanced culture before clinical use.

To assess the in vivo safety, toxicity and biodistribution of thrombin‐preconditioned human Wharton's jelly‐derived mesenchymal stem cells (th‐hWJMSCs) used for neonatal HIE treatment, we performed all preclinical studies as follows: a 6‐month tumorigenicity study in immunodeficient nude mice, an in vivo biodistribution study in a disease model using real‐time PCR and a subchronic general toxicity study in neonatal Sprague‐Dawley (SD) rats. Because it is a stem cell treatment for newborns, the evaluation of developmental behaviour was included in the general toxicity study. In addition, in order to evaluate the possibility of excessive immunosuppression by overdose and changes in the expression patterns of histocompatibility antigens, changes in immune cells in blood were evaluated. In line with the preclinical study that demonstrated the therapeutic efficacy of hWJMSCs in the neonatal HIE rat pup model,^16^ herein, we administered hWJMSCs intracerebroventricularly. The studies were designed based on “Guideline in Quality, Non‐clinical and Clinical Assessment of Stem Cell Therapy Product” of the Ministry of Food and Drug Safety (MFDS) in the Republic of Korea (https://www.mfds.go.kr).

## 2 MATERIALS AND METHODS

### 2.1 Test article

The procedure for cell culture was approved by the Institutional Review Board (IRB) of the Samsung Medical Center. The th‐hWJMSCs were prepared at the Good Manufacturing Practice facility of the Samsung Stem Cells and Regenerative Medicine Institute (SSCRMI). Human Wharton's jelly‐derived mesenchymal stem cells (hWJMSCs) were isolated and expanded as described previously, after obtaining consent from pregnant mothers.^19^ hWJMSCs from a single donor at passages 5–6 were used in this study. The stemness of the hWJMSCs was confirmed using in vitro differentiation assays for osteogenesis, adipogenesis, and chondrogenesis and flow‐cytometric analysis of cell surface markers (CD73, CD90, CD105, CD166, CD14, CD11b, HLA‐DR (MHCII), CD34, CD45 and CD19), as described previously.^20^ After reaching more than 90% confluence, the hWJMSCs were preconditioned with thrombin (2 U/mL; Sigma‐Aldrich, Steinheim, Germany) in culture medium (α‐MEM; Gibco, Life Technologies) for 3 h as per an optimal preconditioning regimen protocol.^21^ The th‐hWJMSCs were appropriate in appearance and microbial sterility (48 h). Further, the th‐hWJMSCs were deemed suitable for use in the mycoplasma test, adventitious virus contamination test and endotoxin test, and the cell viability was over 90%. The th‐hWJMSCs used in these studies were supplied as ready‐to‐use formulations counted using the Luna‐FL™ system (Logos Biosystems) on the day of administration by SSCRMI. In the tumorigenicity study, human uppsala 87 malignant glioma (U87MG) cells, which were prepared at the Korea Institute of Toxicology (KIT), were used as positive control materials.^22^

### 2.2 Dose selection

In the in vivo studies, the highest dose of th‐hWJMSCs was 1 × 10^5^ cells/10 µL/head, the maximum feasible dose, based on the Guidance on Nonclinical Safety Studies for the Conduct of Human Clinical Trials and Marketing Authorization for Pharmaceuticals M3 (R2).^23^ In addition, the additional one or two lower doses were selected to define a dose‐dependent response on any findings observed at the highest dose in the tumorigenicity and general toxicity studies. The vehicle control group was administered the same culture medium.

In the tumorigenicity test, the positive control material, U87MG cells, was administered in the mouse cerebral ventricle via ICV injection at 3 × 10^4^ cells/10 µL/head based on the preliminary test. In the preliminary test, ICV injection was administered to six male mice. It was confirmed that all animals died within 2 months at 3 × 10^4^ cells/10 µL/head, and tumours were formed in their brain tissues.

### 2.3 Animal husbandry and maintenance

The subchronic general toxicity study, in vivo tumorigenicity study and biodistribution studies were performed with rodent species purchased from Orient Co. The animals were housed in a room maintained at a temperature of 23 ± 3°C, relative humidity of 50 ± 10% with artificial lighting from 08:00–20:00, and 13–18 air changes per hour. The animals were housed socially in polysulfone cages with Aspen chip bedding (Tapvei Estonia OÜ, Paekna, Estland) and allowed access to sterilized tap water and commercial rodent chow (Lab Diet® #5053; PMI Nutrition International) ad libitum. This experiment was conducted in facilities approved by the Association for Assessment and Accreditation of Laboratory Animal Care International (AAALAC). All animal protocols were reviewed by the Institutional Animal Care and Use Committee of KIT or the Samsung Medical Center.

### 2.4 Surgical procedure and ICV injection

The surgical procedure and ICV injection techniques were adapted from Schmidt's method, with minor modifications.^24^ Under deep isoflurane anaesthesia, the animals were placed in the stereotaxic frame. After making a midline incision from the frontal cranial bones, th‐hWJMSCs were administered using an infusion pump with 31G insulin syringe into the area of AP (Anterior‐Posterior) −0.50, ML (Medial‐Laeral) −1.15, and DV (Dorsal‐Ventral) −2.70 mm in neonatal SD rats and AP −0.50, ML −1.20, and DV −2.40 mm in BALB/c nude mice from Bregma. The stereotaxic coordinates were determined using preliminary experiments. The administration was performed at approximately 10 µL/min and then slowly withdrawn after a certain time (more than 1 min). The animals were sutured and placed in a thermostat for recovery until the influence of anaesthesia wore off.

### 2.5 Subchronic general toxicity study in neonatal SD rats

The subchronic general toxicity study was conducted under Good Laboratory Practice (GLP) regulations of Organisation for Economic Co‐operation and Development (OECD)^25^ and US Food and Drug Administration (FDA).^26^ The study was designed in accordance with the International Conference on Harmonization (ICH) Harmonized Tripartite Guidelines S5 (R2), Detection of Toxicity to Reproduction for Medicinal Products & Toxicity to Male Fertility (2005)^27^ and European Medicines Agency (EMA) Guidelines concerning the Need for Non‐clinical Testing in Juvenile Animals on Human Pharmaceuticals for Pediatric Indications (2008).^28^

Postnatal day 5 (PND 5) SD rats were assigned to four treatment groups (15 animals/sex/group). The th‐hWJMSCs were administered to the rats once by ICV injection at dose levels of 0 (vehicle control), 1 × 10^4^, 3 × 10^4^ and 1 × 10^5^ cells/10 µL/head on PND 7 when the rodent brain corresponded to 36–40 weeks of gestation in humans.^29^ Observations for mortality, clinical signs, body weight and food consumption were recorded for eight weeks. Five animals/sex/group were euthanized using isoflurane at week 4, and then macroscopic observations and organ weight measurements were conducted. Clinical pathology assessments including haematology, coagulation, clinical chemistry, urinalysis and urine chemistry were conducted prior to sacrifice. To analyse the changes in immune cells in the blood, blood samples were collected into EDTA‐2K tubes. Further, the cells were single‐or double‐stained with anti‐rat CD3, CD4, CD8, CD45RA and CD161a antibodies (all from BD Biosciences). The changes in cell percentage (%) and CD4:CD8 T‐cell ratio were confirmed by flow cytometry (FACS Calibur; BD Biosciences).

In addition, histopathological examinations were conducted for the full list of tissues. In addition, impacts on growth including physical development, behavioural function, learning and memory, and sexual maturation were assessed in the last 10 animals/sex/group until week 8. To assess physical development, the dates of completion of fur formation (PND 8 to completion), incisor eruption (PND 10 to completion) and eyelid opening (PND 12 to completion) were observed daily and recorded. The behavioural function tests included negative geotaxis test (PND 10 to completion), traction test (PND 15 to completion), acoustic startle response (PND 22 to completion), pupillary reflex (PND 22 to completion), rotating rod test (PND 23 to completion) and motor activity test (PND 39–45). A Morris water maze test was conducted from PND 60–66 for learning and memory evaluation. In addition, sexual maturation completion dates including of vaginal opening (PND 26 to completion) and preputial separation (PND 36 to completion) were observed for the newborn animals.

### 2.6 In vivo Biodistribution study in disease model

The in vivo biodistribution study was designed based on the Considerations in Biodistribution Assessment of Stem Cell Therapy Product of Korea MFDS^30^ and published literature.^31^

The HIE disease model in PND 7 SD rats was induced by ligation of the unilateral (right side) carotid artery, followed by exposure to 8% oxygen for 2 h in 35 males and 35 females, including sham controls at the Samsung Medical Center. The animals were evaluated for sufficient brain damage by checking the infarction area using brain diffusion‐weighted imaging. After administration of th‐hWJMSCs at a dose of 1 × 10^5^ cells/10 µL/head into the cerebral ventricle of the HIE disease animals, 5 animals/sex/timepoint were provided to KIT to collect organ samples on each scheduled sacrifice day (1, 2, 4, 7, 28 and 91 days after administration). The sham controls were sacrificed on day 1, without any administration.

After sacrifice, the human *Alu* gene expression was measured from the collected organs (the brain, spinal cord, blood, heart, lung, liver, spleen, kidney, mesenteric lymph node, pancreas, epididymis, ovary, testis and uterus) using real‐time PCR (Applied Biosystems ViiA™ 7 Real‐Time PCR System) from all animals, and the results were analysed using the analysis software (ViiA RUO S/W v 1.2.2). The validated quantitative conditions for the analysis of the human *Alu* gene in SD rat tissue using real‐time PCR are shown in Table S1.

### 2.7 Tumorigenicity study in nude mice

The tumorigenicity study was conducted under GLP regulations of OECD^25^ and Korea MFDS^32^ and designed in accordance with ICH Guideline Specification: Test Procedure and Acceptance Criteria for Biotechnological/Biological Products, Q6B, Step 4,^33^ and Guideline on Tumorigenicity Assessment of Stem Cell Therapy Products of Korea MFDS.^34^

Four‐week‐old BALB/c nude mice were assigned to four treatment groups (13 animals/sex/group) and kept in the animal room for 1 week. Four‐week‐old mice were the youngest of the nude mice available for purchase. The th‐hWJMSCs were administered once by ICV injection at dose levels of 0 (vehicle control), 3 × 10^4^ and 1 × 10^5^ cells/10 µL/head at 5 weeks of age. To compare tumour formation, U87MG cells as a positive control were administered with the same regimen at a dose level of 3 × 10^4^ cells/10 µL/head into the positive control group. Observations for mortality, clinical signs including the presence of tumours and body weight were recorded for 26 weeks. Haematology was conducted prior to necropsy, except in the positive control group. All animals were euthanized using isoflurane at the end of the study period, and macroscopic observations and histopathological examinations of the liver with the gall bladder, adrenal glands, lungs with bronchi, mandibular lymph node, brain (injection site), mesenteric lymph node, heart, spleen, kidneys and stomach were conducted. In addition, immunohistochemistry to assure accurate analysis of tumour origin was performed using a cell‐specific marker, anti‐human mitochondria antibody (ab92824; Abcam) and anti‐mouse cyclophilin A (D2Y4 M; Cell Signaling Technology) for the tumour tissues.^35^

### 2.8 Statistical analysis

Multiple comparison tests for different dose groups were conducted using the means and standard deviations from each group. Variance homogeneity was assessed using the Bartlett's test. Homogeneous data were assessed using one‐way analysis of variance (ANOVA), and significant differences between some groups were assessed using Dunnett's test. Heterogeneous data were assessed using the Kruskal‐Wallis test, and significant differences between the control and treated groups were assessed using Dunn's rank‐sum test. Categorical data were assessed using the χ^2^‐test followed by Fisher's exact test. For comparison between two groups, variance homogeneity was assessed using the F‐test. Homogeneous data were assessed using Student's *t* test, while heterogeneous data were assessed using the Wilcoxon rank‐sum test. Statistical analyses were conducted automatically using the Pristima System (Version 7.3; Xybion Medical Systems). The results of the comparison were considered significant when the *p* values were less than 0.05 or 0.01.

## 3 RESULTS

### 3.1 Subchronic general toxicity study in neonatal SD rats

**FIGURE 1:**
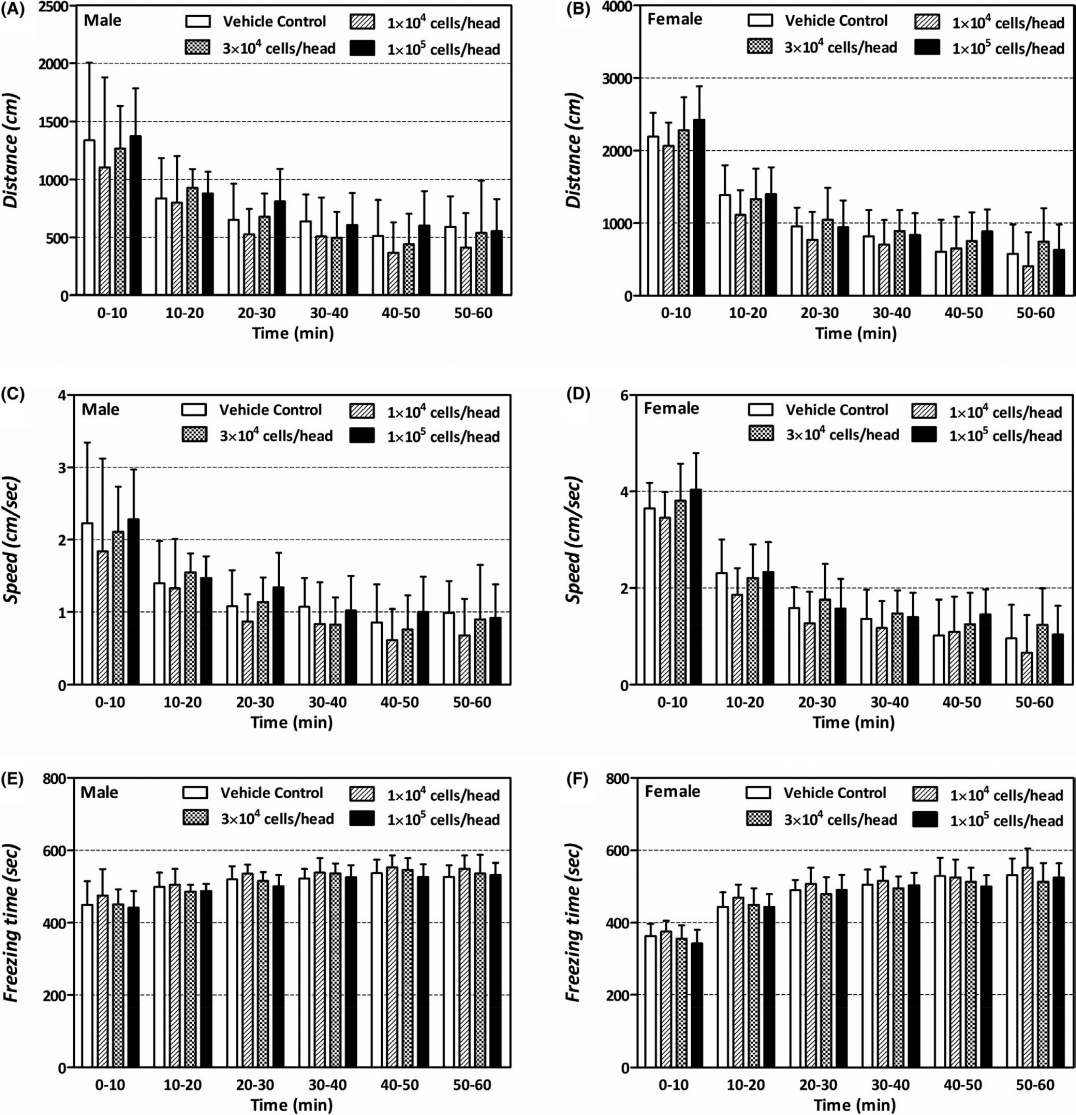
Comparison of motor activity between the four groups in subchronic general toxicity study. The motor activity on PND 39–45 was measured every 10 min for 1 h with Etho Vision XT Version 8.5 (Noldus Information Technology B.V., Wageningen, Netherlands). (A, B): mean distanced moved (cm). (C, D) mean moving speed (cm/sec). (E, F) mean freezing time (sec)

No mortality or obvious clinical signs related to the th‐hWJMSCs were observed during the study period. In addition, no abnormal changes were observed in body weight and food consumption, clinical pathology, macro/microscopic observation, lymphocyte analysis, and developmental evaluation, including growth pattern, behaviour, learning and memory function, and sexual maturity in the th‐hWJMSC‐treated groups. The results are presented in Tables 1, 2, 3, Tables S2 and S3 and Figure 1.

**TABLE 1:** Comparison of physical development among the four groups in subchronic general toxicity study

Parameters	0 cells/head	1 × 10^4^ cells/head	3 × 10^4^ cells/head	1 × 10^5^ cells/head
	Sex: Male			
Fur development	8.00 ± 0.00	8.00 ± 0.00	8.00 ± 0.00	8.00 ± 0.00
Incisor eruption	10.10 ± 0.32	10.50 ± 0.53	10.30 ± 0.48	10.30 ± 0.48
Eyelid opening	13.90 ± 0.32	13.90 ± 0.57	13.60 ± 1.07	13.70 ± 0.82
	Sex: Female			
Fur Development	8.00 ± 0.00	8.00 ± 0.00	8.00 ± 0.00	8.00 ± 0.00
Incisor Eruption	10.10 ± 0.32	10.20 ± 0.42	10.10 ± 0.32	10.40 ± 0.52
Eyelid Opening	13.60 ± 0.70	13.80 ± 0.42	13.70 ± 0.82	13.60 ± 0.70

Note

Values are post‐natal day (PND) mean ± standard error of 10 rats/sex/group. The evaluation of fur development, incisor eruption and eyelid opening began on PND 8, PND 10 and PND 12, respectively.

**TABLE 2:** Comparison of behavioural function test among the four groups in subchronic general toxicity study

Parameters	0 cells/head	1 × 10^4^ cells/head	3 × 10^4^ cells/head	1 × 10^5^ cells/head
	Sex: Male			
Negative geotaxis	10.10 ± 0.32	10.20 ± 0.42	10.00 ± 0.00	10.00 ± 0.00
Traction test	15.30 ± 0.48	15.80 ± 1.03	15.70 ± 0.95	16.00 ± 0.94
Pupillary reflex	22.00 ± 0.00	22.00 ± 0.00	22.00 ± 0.00	22.00 ± 0.00
Acoustic startle response	22.00 ± 0.00	22.00 ± 0.00	22.00 ± 0.00	22.00 ± 0.00
Rotating rod test	23.50 ± 0.71	23.60 ± 0.84	23.30 ± 0.67	23.60 ± 0.70
	Sex: Female			
Negative geotaxis	10.10 ± 0.32	10.00 ± 0.00	10.00 ± 0.00	10.20 ± 0.42
Traction test	16.30 ± 1.64	15.80 ± 1.32	16.00 ± 1.33	15.70 ± 0.82
Pupillary reflex	22.00 ± 0.00	22.00 ± 0.00	22.00 ± 0.00	22.00 ± 0.00
Acoustic startle response	22.00 ± 0.00	22.00 ± 0.00	22.00 ± 0.00	22.00 ± 0.00
Rotating rod test	23.30 ± 0.48	23.20 ± 0.42	23.60 ± 0.70	24.10 ± 0.74

Note

Values are post‐natal day (PND) mean ± standard error of 10 rats/sex/group. The evaluation of negative geotaxis, traction test, acoustic startle response, pupillary reflex and rotating rod test began on PND 10, PND 15, PND 22 and PND 22, respectively.

**TABLE 3:** Comparison of lymphocyte phenotyping analysis among the four groups in subchronic general toxicity study

Cell population (Phenotype)	0 cells/head	1 × 10^4^ cells/head	3 × 10^4^ cells/head	1 × 10^5^ cells/head
	Sex: Male			
Total T cells (CD3+)	52.26 ± 4.85	51.14 ± 6.46	49.18 ± 3.87	52.47 ± 2.24
Th cells (CD3+ CD4+)	37.51 ± 4.45	34.72 ± 5.83	34.41 ± 3.41	36.23 ± 2.37
Tc cells (CD3+ CD8+)	14.71 ± 1.09	16.94 ± 1.22^a^	14.91 ± 0.86	16.35 ± 1.36
Ratio (CD4:CD8)	2.56	2.05	2.31	2.23
B cells (CD3− CD45RA+)	45.18 ± 4.67	42.96 ± 5.38	46.87 ± 3.53	44.70 ± 2.91
NK cells (CD3‐ CD161a+)	0.97 ± 0.22	0.95 ± 0.30	0.82 ± 0.23	0.89 ± 0.22
	Sex: Female			
Total T cells (CD3+)	55.13 ± 8.24	55.25 ± 8.17	54.28 ± 5.51	55.62 ± 4.86
Th cells (CD3+ CD4+)	39.87 ± 4.71	40.05 ± 5.48	39.18 ± 4.62	38.45 ± 3.91
Tc cells (CD3+ CD8+)	15.48 ± 4.09	15.43 ± 3.89	15.24 ± 3.42	17.29 ± 2.23
Ratio (CD4:CD8)	2.71	2.71	2.73	2.25
B cells (CD3− CD45RA+)	41.63 ± 7.45	41.19 ± 7.13	42.83 ± 4.90	40.83 ± 5.28
NK cells (CD3− CD161a+)	1.12 ± 0.28	1.09 ± 0.33	1.42 ± 0.69	1.02 ± 0.15

Note

Values are percentage mean ± standard error of 5 rats/sex/group. The lymphocyte phenotyping analysis was conducted on PND 57.

Tc cells, cytotoxic T cells; Th cells, helper T cells.

^a^ Dunnett's test significant at the 0.05 level.

A significant decrease in absolute basophil count and increase in inorganic phosphorus were observed in females at 1 × 10^5^ cells/10 µL/head. In addition, significant increases in absolute and relative (over brain and terminal body weight) weights of the thyroid were observed in females at more than 3 × 10^4^ cells/10 µL/head (data not shown). However, histopathological examination did not reveal any associated lesions.

Based on these results, no adverse effect level (NOAEL) of th‐hWJMSCs was considered at 1 × 10^5^ cells/10 µL/head in newborn SD rats (PND 7) under the condition of a single ICV administration.

### 3.2 Biodistribution in disease model

No abnormal changes or lesions were observed in the animals with HIE disease during the study period. Approximately, 840 samples from the collected organs were analysed using the established method.

**FIGURE 2:**
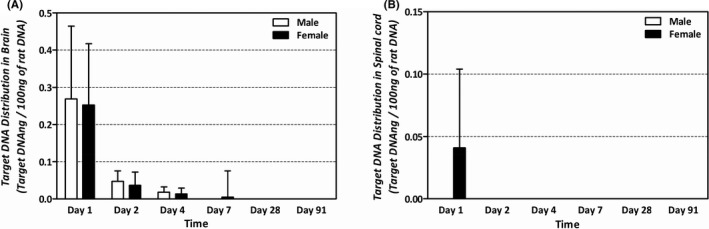
Tissue distribution of human *Alu* gene determined with real‐time PCR after ICV injection of 1 × 10^5^ cells/10 µL/head in HIE disease animals. (A) Relative mean number of copies of human Alu gene in brain of rats. (B) Relative mean number of copies of human Alu gene in spinal cord of rats. No human Alu gene was detected in the spinal cord of male rats from Day 1

The analysis revealed that the human *Alu* gene expression in th‐hWJMSC‐treated male mice was detected on days 1, 2 and 4 after administration in the brain, and it decreased over time (0.269, 0.047 and 0.018 ng/100 ng of rat DNA). The human *Alu* gene expression of th‐hWJMSC‐treated female mice was detected on days 1, 2, 4 and 7 in the brain and decreased over time, similar to males (0.252, 0.036, 0.013 and 0.005 ng/100 ng of rat DNA) (Figure 2A). In females, trace expression levels of human *Alu* gene (0.041 ng/100 ng of rat DNA) were detected on day 1 in the spinal cord, but not thereafter. In males, human *Alu* gene was not detected in the spinal cord (Figure 2B). The gene expression in other tissues (blood, heart, lung, liver, spleen, kidney, mesenteric lymph nodes, pancreas, testes, epididymis, ovaries and uterus) was not detected in either sex.

### 3.3 Tumorigenicity study in nude mice

No abnormal changes were observed in clinical signs, body weight or haematology in the th‐hWJMSC‐treated groups. No tumours were observed in the brain at the site of administration.

**FIGURE 3:**
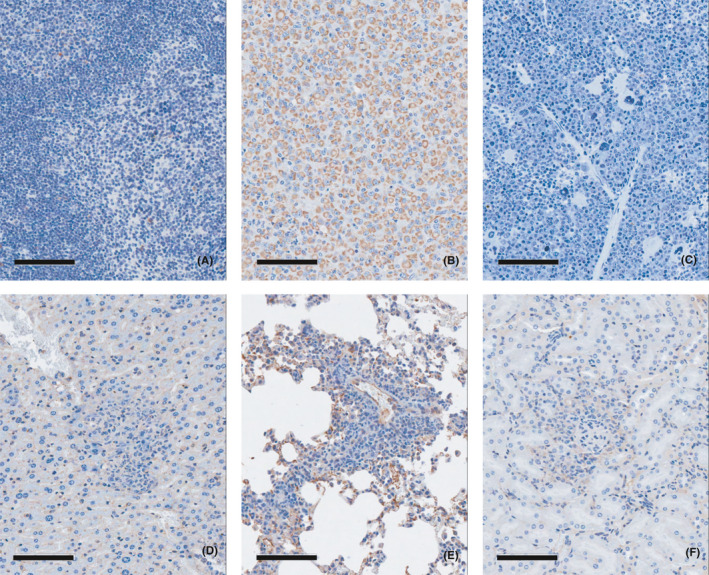
Immunohistochemistry in the lymphoma tissues with a human‐specific anti‐mitochondria antibody in tumorigenicity study. (A) Negative control tissue (spleen), (B) Positive control tissue (U87MG injection site, brain), (C) Lymphoma tissue (spleen), (D) Lymphoma tissue (liver), (E) Lymphoma tissue (lung), (F) Lymphoma tissue (kidney). Human‐specific anti‐mitochondria antibody showed positively stained U87MG tumour cells in brain of the positive control tissue (B). On the other hand, negative results for anti‐mitochondria antibody were observed from the malignant lymphoma tissues (C, D, E, F)

**FIGURE 4:**
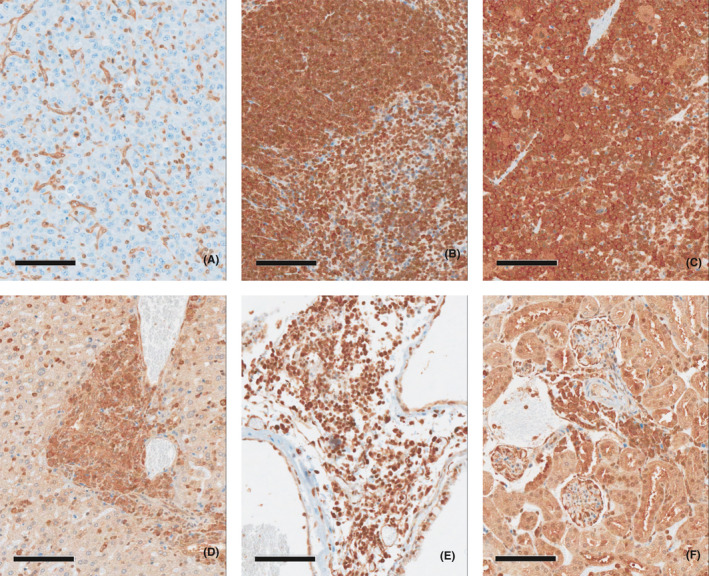
Immunohistochemistry in the lymphoma tissues with a mouse‐specific anti‐cyclophilin A antibody in tumorigenicity study. (A) Negative control tissue (U87MG injection site, brain), (B) Positive control tissue (spleen), (C) Lymphoma tissue (spleen), (D) Lymphoma tissue (liver), (E) Lymphoma tissue (lung), (F) Lymphoma tissue (kidney). Mouse‐specific anti‐cyclophilin A antibody showed negatively stained U87MG tumour cells in brain (B). On the other hand, anti‐cyclophilin A antibody showed positively stained the malignant lymphoma tissues (C, D, E, F)

One female mouse at a dose of 1 × 10^5^ cells/10 µL/head was found dead on day 106, and enlargement of the spleen and lymph nodes was noted on macroscopic observation. Histopathological examination confirmed abnormal lesions as malignant lymphoma. Additional immunohistochemistry staining for the lymphoma tissues was performed with a human‐specific anti‐mitochondria antibody, which was confirmed to be negative (Figure 3). Conversely, a positive reaction was observed for a procedure involving staining with a mouse‐specific anti‐cyclophilin A antibody (Figure 4).

Malignant tumours transplanted were identified in 10 males and 13 females in the histopathological examination, in the brains of the control group administered U87MG cells at a dose level of 3 × 10^4^ cells/10 µL/head. These results show negligible tumorigenic potential by th‐hWJMSCs at 1 × 10^5^ cells/10 µL/head after a single ICV administration in nude mice.

## 4 DISCUSSION

The incidence of neonatal HIE is 1.5/1000 live births. When ischemia occurs, it often causes irreversible brain damage in many patients.^36^ In other words, HIE is not a disease that can be completely cured through treatment but a disease that requires treatment and management of pathological conditions such as convulsions and neurological disorders from newborn to adulthood.^37^ This means that the disease creates a huge social and economic burden.

Research and clinical trials on HIE treatment using stem cells are ongoing worldwide. In the United States of America, the phase I clinical trial of autologous umbilical cord blood cells transplantation for HIE in neonates was conducted (ClinicalTrials.gov Identifier NCT00593242), and in South Korea, the phase 1 clinical trial of allogeneic Wharton's jelly‐derived MSCs transplantation for HIE in newborns was started in 2020 (CRIS: KCT0004851). Moreover, in Japan, Phase II clinical trials are underway to demonstrate the efficacy of the aforementioned treatments by transplanting autologous cord blood cells into newborns with HIE since 2016 (ClinicalTrials.gov Identifier NCT02256618).

We have been conducting preclinical research on stem cell therapeutics with improved efficacy by preconditioning naïve MSCs with thrombin to overcome the shortcomings of conventional stem cell treatment. The thrombin‐preconditioned MSCs were found to have a significantly higher wound healing effect in an in vivo skin wound model than conventional naïve MSCs.^21^ In the efficacy study of th‐hWJMSCs using a newborn rodent model with severe brain damage, the combination of hypothermia therapy and stem cell therapy induced a decrease in brain cell death and inflammation, along with a reduction in brain damage area and improvement of sensorimotor function.^16^ These results suggest that stem cells maximize their paracrine potency because of thrombin preconditioning, thereby enhancing their therapeutic efficacy.^16^ We conducted this preclinical study to evaluate the potential toxicity of th‐hWJMSCs, which may be caused by the increased paracrine property after thrombin preconditioning, and to assess their tissue distribution to support human clinical trials. In the in vivo tumorigenicity study, immunodeficient BALB/c nude mice were selected as experimental animals in order to minimize immune system rejection against th‐hWJMSCs. However, because neonatal rats have an immature immune system that does not require immunosuppression, they were used for in vivo biodistribution and general toxicity studies without any immunosuppressive agent for the administration of th‐hWJMSCs.^38^, ^39^

MSCs exert immunomodulatory effects on the naïve T cells, helper T cells and cytotoxic T cells.^40^ To analyse changes in immune cells, the percentage (%) and CD4:CD8 T‐cell ratio for each lymphocyte subset were generated using flow cytometry, which is conventionally used for the evaluation of immune cell phenotypes in rodent tests.^41^, ^42^

On day 50 after administration, a statistically significant increase in the number of cytotoxic T cells was observed only in males at 1 × 10^4^ cells/10 µL/head. However, it was not considered to be related to th‐hWJMSCs because no dose dependence was observed, and it was within the range of cytotoxic T‐cell number in normal rats (14%).^43^ In females, no significant changes were observed in any of the immune cells, including cytotoxic T cells. The CD4:CD8 T‐cell ratio was calculated as the percentage (%) of helper T cells (CD3+CD4+) and cytotoxic T cells (CD3+CD8+). Comparing the ratio between helper T cells and cytotoxic T cells, both male and female values were in the range of normal rats (CD4:CD8 T cell ratio = 2–4).^44^ In addition, when compared to the control group, no statistically significant change was observed in either sex.

In the in vivo biodistribution study performed in the HIE disease model of male and female newborns, human *Alu* derived from th‐hWJMSCs was measured, as this approach is useful for detecting human cells among rodent cells.^45^ After administration, the animals were evaluated for the presence and location of the transplanted cells at different time points. It is necessary to examine the potential toxicity and efficacy in relation to the distribution of cells at specific time points.^46^ When th‐hWJMSCs were administered once intracerebroventricularly to HIE disease‐induced newborn rats, they were distributed to the brain from 4 to 7 days and disappeared thereafter. In tissues other than that of the brain, the th‐hWJMSCs were thought to be distributed in small amounts in only the spinal cord for up to 1 day. Therefore, the later distribution profile showing that th‐hWJMSCs were present only in the brain without moving to other organs in the late stage and disappeared within 28 days supports the validity of the toxicity test duration.

As a result of a 26‐week tumorigenicity study in male and female nude mice after a single ICV administration, the tumour‐generating capacity of th‐hWJMSCs was not confirmed. Malignant lymphoma was observed in one female among 26 males and females (approximately 3.8%). However, no positive reaction was observed for lymphoma as a result of immunohistochemical staining using a human cell‐specific anti‐mitochondrial antibody (Figure 3). Conversely, a positive reaction was observed after staining with mouse tissue‐specific anti‐cyclophilin A antibody (Figure 4). Therefore, lymphoma is not considered a tumour caused by the administration of th‐hWJMSCs. In addition, on the microscopic examination after haematoxylin and eosin staining, the findings were histomorphologically similar to those of mouse lymphoma and were considered to be spontaneous changes when considering the incidence of lymphoma in mice.^47^ However, although there is no evidence that th‐hWJMSCs have tumour‐generating capacity in nude mice, it is necessary to evaluate the mechanistic relevance of the paracrine effects on precancerous changes that may occur after 26 weeks. The use of additional in vitro tumorigenicity‐associated tests such as digital soft agar colony formation assays should be considered to detect transformed cells, and RT‐PCR‐based and highly efficient culture methods should be performed to identify residual undifferentiated pluripotent stem cells.^48^

In the present study, the study design of the toxicology evaluation, except for the in vivo biodistribution study, was conducted with healthy animals as the standard test model system. Although immunodeficient healthy rodents can be used for toxicology studies with stem cell therapy products,^49^, ^50^ disease models in the hybrid pharmacology‐toxicology study design may be more appropriate to assess the safety and activity of cell therapeutics because disease model features have potentially prolonged duration, product persistence and complex mode of action in the disease environment.^51^ The use of disease models requires consideration of the risk‐benefit assessment for inherent variability, limited historical data, difficulty in animal care and technical issues with the physiological and anatomical constraints of the model.^51^

In conclusion, the results of the preclinical safety studies indicated that th‐hWJMSCs were not oncogenic, and no abnormal changes or findings related to the thrombin‐preconditioned MSCs were observed. The th‐hWJMSCs were detected in the brain for up to 4 and 7 days in male and female mice, respectively, and in the spinal cord up to the first day for female mice. Therefore, the NOAEL was considered at 1 × 10^5^ cells/10 µL/head under the conditions of this study. This study is expected to provide critical information regarding the clinical use of th‐hWJMSCs and thrombin‐preconditioned cell therapies.

## CONFLICT OF INTEREST

Yun Sil Chang, So Yoon Ahn and Dong Kyung Sung declare potential conflicts of interest arising from a filed patent titled ‘Composition for treating neonatal hypoxic ischemic encephalopathy’ as co‐inventors, not as patentees and this patent was licenced out to MEDINNO INC.

## AUTHOR’S CONTRIBUTIONS

**Jung‐Ho Noh:** Conceptualization (equal); Data curation (lead); Formal analysis (lead); Investigation (equal); Methodology (equal); Visualization (lead); Writing‐original draft (lead); Writing‐review & editing (equal). **Ji‐Seong Jeong:** Formal analysis (supporting); Investigation (equal); Methodology (equal). **Sang‐Jin Park:** Formal analysis (supporting); Investigation (equal); Methodology (equal). **Kyung Jin Jung:** Formal analysis (supporting); Investigation (supporting). **Byoung‐Seok Lee:** Formal analysis (supporting); Investigation (supporting). **Woo‐Jin Kim:** Formal analysis (supporting); Investigation (supporting). **Ji‐Seok Han:** Formal analysis (supporting); Investigation (supporting). **Min‐Kyung Cho:** Visualization (supporting). **Dong Kyung Sung:** Methodology (supporting). **So Yoon Ahn:** Methodology (supporting). **Yun Sil Chang:** Funding acquisition (lead); Methodology (supporting). **Hwa‐Young Son:** Formal analysis (supporting); Investigation (supporting); Methodology (supporting). **Eun Ju Jeong:** Conceptualization (equal); Funding acquisition (supporting); Investigation (supporting); Methodology (supporting); Project administration (lead); Supervision (lead); Writing‐review & editing (equal).

## Supporting information

Table S1

Table S2

Table S3

## Data Availability

The data that support the findings of this study are available from the corresponding author upon reasonable request.
