# Supplementary material for: Preclinical assessment of thrombin‐preconditioned human Wharton’s jelly‐derived mesenchymal stem cells for neonatal hypoxic‐ischaemic brain injury
Source: J Cell Mol Med. 2021 Oct 15;25(22):10430–40. doi: 10.1111/jcmm.16971 (PMC8581315; doi:10.1111/jcmm.16971)
Supplement: Supplementary file 1 — Table S1 [file JCMM-25-10430-s002.docx]

**Supplemental Table 1.** Quantitative conditions for analysis of human *Alu* gene in SD rat tissue using real-time PCR.

| Analytical Method Validation Result | |
| --- | --- |
| Forward primer | 5’- GTC AGG AGA TCG AGA CCA TCC C - 3’ |
| Reverse primer | 5’- TCC TGC CTC AGC CTC CCA AG - 3’ |
| Specificity | No interference |
| Linearity | Calibration standards: 0.005 – 100 ng  Mean curve equation: y = -3.236x + 17.376 (n = 3)  Mean curve correlation: R2 = 0.999 (n =3) |
| Detection limit | 0.0005 ng |
| Lower limit of quantification | 0.005 ng |
| Upper limit of quantification | 100 ng |
| Accuracy (% RE) | HQC: -8.18, MQC: 6.32, LQC: -11.12, LLOQ: -9.40 |
| Precision (Repeatability, % RSD) | HQC: 4.85, MQC: 3.30, LQC: 2.59, LLOQ: 3.14 |
| Precision (Different day, % RSD) | HQC: 5.47, MQC: 3.79, LQC: 3.34, LLOQ: 4.28 |
| Precision (Different instrument, % RSD) | HQC: 7.28, MQC: 4.89, LQC: 4.25, LLOQ: 3.99 |
| Precision (Different analyst, %RSD) | HQC: 4.16, MQC: 11.85, LQC: 3.41, LLOQ: 4.00 |
| \| HQC \| High Quality Control, 80 ng \| \| \| \| --- \| --- \| --- \| --- \| \| LLOQ \| Lower Limit of Quantification, 0.005 ng \| \| \| \| LQC \| Low Quality Control, 0.015 ng, \| \| \| \| MQC \| Middle Quality Control, 0.7 ng \| \| \| \| QC \| Quality Control \| \| \| \| ULOQ \| Upper Limit of Quantification \| \| \| \| %RSD \| Relative Standard Deviation \| \| \| \| %RE \| Percent Relative Error \| \| \| \| %RE = \| (Mean value of samples - Nominal value ) x 100 \|  \| \| \| Nominal concentration \| \| %RSD= \| Standard deviation for concentration of samples x 100 \| \|  \| \| Mean concentration of samples \| \| | |
