# Supplementary material for: Preclinical assessment of thrombin‐preconditioned human Wharton’s jelly‐derived mesenchymal stem cells for neonatal hypoxic‐ischaemic brain injury
Source: J Cell Mol Med. 2021 Oct 15;25(22):10430–40. doi: 10.1111/jcmm.16971 (PMC8581315; doi:10.1111/jcmm.16971)
Supplement: Supplementary file 2 — Table S2 [file JCMM-25-10430-s003.docx]

**Supplemental Table 2.** Comparison of Morris Water Maze test among the four groups in subchronic general toxicity study

|  | Sex: Male | | | |
| --- | --- | --- | --- | --- |
| Test Day | 0 cells/head | 1×10^4^ cells/head | 3×10^4^ cells/head | 1×10^5^ cells/head |
| First Day |  |  |  |  |
| 1^st^ trial | 60.76 ± 36.60 | 78.22 ± 19.14 | 82.87 ± 20.68 | 83.96 ± 11.76 |
| 2^nd^ trial | 65.82 ± 30.71 | 64.99 ± 32.44 | 68.92 ± 30.63 | 61.21 ± 33.85 |
| 3^rd^ trial | 44.81 ± 36.26 | 64.08 ± 27.46 | 48.07 ± 38.20 | 36.25 ± 24.04 |
|  |  |  |  |  |
| Second Day |  |  |  |  |
| 1^st^ trial | 53.73 ± 33.11 | 44.35 ± 33.33 | 69.12 ± 30.41 | 49.73 ± 33.62 |
| 2^nd^ trial | 29.63 ± 15.01 | 35.13 ± 21.43 | 40.18 ± 35.42 | 33.42 ± 25.30 |
| 3^rd^ trial | 28.87 ± 19.86 | 39.77 ± 21.72 | 26.19 ± 23.62 | 30.25 ± 24.63 |
|  |  |  |  |  |
| Third Day |  |  |  |  |
| 1^st^ trial | 47.37 ± 35.73 | 30.90 ± 25.75 | 36.36 ± 30.86 | 47.16 ± 36.11 |
| 2^nd^ trial | 33.70 ± 27.70 | 34.86 ± 27.44 | 26.86 ± 22.40 | 22.16 ± 12.32 |
| 3^rd^ trial | 27.76 ± 28.32 | 28.54 ± 29.08 | 15.74 ± 13.25 | 32.53 ± 21.82 |
|  | Sex: Female | | | |
| Test Day | 0 cells/head | 1×10^4^ cells/head | 3×10^4^ cells/head | 1×10^5^ cells/head |
| First Day |  |  |  |  |
| 1^st^ trial | 75.72 ± 23.25 | 66.27 ± 32.67 | 60.18 ± 30.26 | 65.12 ± 32.22 |
| 2^nd^ trial | 46.71 ± 32.28 | 37.45 ± 29.49 | 54.76 ± 35.66 | 65.65 ± 26.68 |
| 3^rd^ trial | 46.10 ± 29.51 | 40.81 ± 33.30 | 42.87 ± 33.78 | 32.00 ± 28.65 |
|  |  |  |  |  |
| Second Day |  |  |  |  |
| 1^st^ trial | 46.01 ± 34.21 | 42.65 ± 30.24 | 30.79 ± 19.31 | 59.61 ± 34.54 |
| 2^nd^ trial | 37.04 ± 35.85 | 37.50 ± 24.72 | 27.49 ± 31.16 | 36.37 ± 33.83 |
| 3^rd^ trial | 34.56 ± 31.75 | 29.80 ± 31.44 | 27.57 ± 24.11 | 40.00 ± 32.80 |
|  |  |  |  |  |
| Third Day |  |  |  |  |
| 1^st^ trial | 49.96 ± 34.42 | 28.04 ± 28.26 | 44.70 ± 38.68 | 42.61 ± 35.32 |
| 2^nd^ trial | 23.94 ± 21.46 | 42.57 ± 26.52 | 42.21 ± 34.09 | 37.72 ± 32.05 |
| 3^rd^ trial | 14.77 ± 13.40 | 43.24 ± 28.76* | 22.96 ± 15.27 | 26.54 ± 25.14 |

Values are sec mean ± standard error of 10 rats/sex/group. The water maze test was conducted between PND 60 and 66.

*: Dunnett’s test significant at the 0.05 level.
