# Supplementary material for: Preclinical assessment of thrombin‐preconditioned human Wharton’s jelly‐derived mesenchymal stem cells for neonatal hypoxic‐ischaemic brain injury
Source: J Cell Mol Med. 2021 Oct 15;25(22):10430–40. doi: 10.1111/jcmm.16971 (PMC8581315; doi:10.1111/jcmm.16971)
Supplement: Supplementary file 3 — Table S3 [file JCMM-25-10430-s001.docx]

**Supplemental Table 3.** Comparison of sexual maturation among the four groups in subchronic general toxicity study

|  | Sex: Male | | | |
| --- | --- | --- | --- | --- |
| Parameters | 0 cells/head | 1×10^4^ cells/head | 3×10^4^ cells/head | 1×10^5^ cells/head |
| Preputial Separation (Day) | 42.30 ± 1.06 | 41.40 ± 1.35 | 41.20 ± 1.03 | 41.70 ± 1.42 |
| Body Weight on Preputial Separation (g) | 240.57 ± 13.92 | 244.16 ± 19.94 | 238.52 ± 14.64 | 234.49 ± 14.56 |
|  | Sex: Female | | | |
| Parameters | 0 cells/head | 1×10^4^ cells/head | 3×10^4^ cells/head | 1×10^5^ cells/head |
| Vaginal Opening (Day) | 31.50 ± 1.27 | 31.50 ± 0.97 | 31.70 ± 1.06 | 30.90 ± 1.79 |
| Body Weight on Vaginal Opening (g) | 120.91 ± 8.77 | 124.29 ± 7.78 | 122.69 ± 13.88 | 120.77 ± 18.27 |

Values are mean ± standard error of 10 rats/sex/group. The evaluation of preputial separation and vaginal opening began on PND 36 and PND 26, respectively.
